# The missed lessons of Sir Austin Bradford Hill

**DOI:** 10.1186/1742-5573-1-3

**Published:** 2004-10-04

**Authors:** Carl V Phillips, Karen J Goodman

**Affiliations:** 1 Management, Policy and Community Health Division, University of Texas School of Public Health, 1200 Pressler, Houston, TX 77225, USA; 2 Center for Clinical Research and Evidence-Based Medicine, University of Texas Medical School, Houston, TX USA; 3 Center for Philosophy, Health, and Policy Sciences, Inc, Houston, USA

**Keywords:** epidemiologic methods, health policy, causal inference

## Abstract

Austin Bradford Hill's landmark 1965 paper contains several important lessons for the current conduct of epidemiology. Unfortunately, it is almost exclusively cited as the source of the "Bradford-Hill criteria" for inferring causation when association is observed, despite Hill's explicit statement that cause-effect decisions cannot be based on a set of rules. Overlooked are Hill's important lessons about how to make decisions based on epidemiologic evidence. He advised epidemiologists to avoid over-emphasizing statistical significance testing, given the observation that systematic error is often greater than random error. His compelling and intuitive examples point out the need to consider costs and benefits when making decisions about health-promoting interventions. These lessons, which offer ways to dramatically increase the contribution of health science to decision making, are as needed today as they were when Hill presented them.

## Introduction

One of the most cited papers in health research is Austin Bradford Hill's "The Environment and Disease: Association or Causation?" [1], Hill's 1965 Presidential Address to the Section of Occupational Medicine of the Royal Society of Medicine, where he presented what are now commonly called the "Bradford-Hill criteria." This paper ironically gains its fame for providing a checklist for inferring causation, something Hill did not claim to be creating. Meanwhile, largely ignored are its great insights and potential contributions to critical methodological and policy issues.

Hill outlined a systematic approach for using scientific judgment to infer causation from statistical associations observed in epidemiologic data, listing nine issues to be considered when judging whether an observed association is a causal relationship. Despite widely distributed and clearly elaborated advice to the contrary [2], Hill's nine considerations are still frequently taught to students of epidemiology and referred to in the literature as "causal criteria." Typically presented as a checklist approach to assessing causation (though without a method for deciding whether to assign a particular checkmark, let alone how to make a final assessment), Hill's list is commonly taught in epidemiology courses and is probably invoked more often than any other method for assessing causation. At a time when the discussion of the nature of causation and methods for identifying causal effects are reaching new levels of sophistication in epidemiology [3-5], this is particularly unfortunate.

Hill never used the term "criteria" and he explicitly stated that he did not believe any hard-and-fast rules of evidence could be laid down, emphasizing that his nine "viewpoints" [1](p. 299) were neither necessary nor sufficient for causation. His suggestions about how to intuitively assess causation are almost completely lost when his address is distilled into a checklist (See endnote 1).

Causal criteria are an intriguing subject for the history of science, including the question of why Hill's list seems more popular than others [7-10] and whether causal conclusions that explicitly appealed to criteria are more likely to be borne out by subsequent evidence. (To our knowledge, there has been no such validation study of causal criteria.) But it is not the main purpose of this analysis to join the extensive discussion of the history and merits of causal criteria. We will say only that Hill's list seems to have been a useful contribution to a young science that surely needed systematic thinking, but it long since should have been relegated to part of the historical foundation, as an early rough cut. Yet it is still being recited by many as something like natural law. Appealing in our teaching and epistemology to the untested "criteria" of a great luminary from the past is reminiscent of the "scientific" methods of the Dark Ages. Hill's own caveats suggest a similar opinion (though such a claim requires some caution, given that Hill repeated his list in his medical statistics textbooks until the time of his death, adding neither an evolution in his perspective nor arguments to support the validity or usefulness of the list [11-14]).

This brief analysis of Hill's "criteria" and what has been made of them can add little new to that topic (though we will argue that Hill deserves more credit than he is usually given by critics of "criteria" for the nuances and examples he presented). Our purpose is to call attention to the seldom-cited last page and a half of the article, which presents lessons that remain overlooked today.

## Analysis

Hill eloquently warned about overemphasis on statistical significance testing, writing "the glitter of the *t *table diverts attention from the inadequacies of the fare" [1](p. 299). The mistake of drawing conclusions from inadequate samples had been replaced with the mistake of treating statistical significance as necessary and sufficient for action. An intellectual generation passed after 1965 with almost no improvement [15], and little has changed in another generation after that. Researchers still frequently present results as if statistical significance and p-values are useful decision criteria, and decision makers are left with inadequate information.

One implication of Hill's advice is well understood. Emphasis on the p-value (let alone dichotomous statements of significance) has been soundly denounced for decades [16,17]. Estimation of effect sizes, presented as point estimates with confidence intervals, is the preferred method in current textbooks [18] and these are generally reported, though in practice confidence intervals tend to be interpreted as mere tests of statistical significance by ignoring their range except to note whether or not they include the null value (see endnote 2).

A further inadequacy of the fare is less well appreciated, stemming not from the question of p-values versus confidence intervals, but from systematic errors. No statistical test of random sampling error informs us about the possible impacts of measurement error, confounding, and selection bias. Methods for quantifying such errors (and perhaps more importantly, arguments for why we need to do so) have been developed in epidemiology, particularly over the last five years [19-25]. Hill hinted at this more than three decades before the recent spate of attention when he noted that one of his own studies [26], like many studies, had great potential for selection bias (though he does not use this term). In effect, he asks "why would I bother to do an exaggeratedly precise statistical test when I know that the other sources of error are likely so large?" Rather than emphasize low p-values, he concluded that simple cell counts made both random error and plausible systematic error unlikely to account for the observed association. While his solution was inadequate – indeed, it might even be called hubris (see endnote 3) – he did issue a clear warning about mistaking statistical precision for validity. Despite the influence of Hill's article, the fact that it contained this point is forgotten (and the point, while obviously true, remains widely ignored).

Even as modern epidemiologic analysts become less dazzled by the t-table, replacing significance testing with confidence intervals and introducing quantification of systematic errors, there is still a tendency to completely overlook Hill's other important insight. Hill sought to address the question how to decide whether to take action once causal inferences are made. In his last few paragraphs, he offers an important commentary on the policy recommendations that flow from decisions regarding cause and effect in epidemiology. Since "our object is usually to take action" [1](p. 300), policy considerations are central to the importance of the science. While epidemiology has its roots in specific policy questions ("can we do something to prevent cholera outbreaks?"), epidemiologists have ambivalent attitudes towards the policy decisions associated with their research [31]. In grant applications and introductions to research reports, it is typical for epidemiologists to justify expensive research based on immediate practical benefits. But in presenting the results, they often deny, implicitly or explicitly, the need to assess the policy contributions [32], defending the value of science for its own sake (sometimes even as they issue press releases calling for policy responses).

Even when policy implications are presented explicitly, they are seldom carefully analyzed. Analyzing the implications of a health research finding for decision making is often not terribly difficult, but making recommendations without such analysis can lead to absurd suggestions [33]. One epidemiology journal famously goes so far as to instruct authors to avoid the common practice of tacking on policy recommendations at the end of research reports. The argument is that policy analysis is too complicated and too serious to be an afterthought by researchers whose expertise lies elsewhere [34,35]. Judging from Hill's comments, he might have preferred more careful policy analysis be included in epidemiologic research reports, rather than none at all, though it is not clear he could solve the challenge of fitting it into the standard 3000-word, single-result health research paper. The present journal offers a solution by publishing policy analyses that are based on health research results, and allowing the articles to be whatever length they need be [36].

Hill, who was educated in economics, argued that in order to take policy action, we ought to pay attention to the absolute costs and benefits of potential actions. It would clearly be reading too much into the text to suggest that he had a prescient vision of modern probability-weighted cost and benefit based policy analyses and decision theory (those fields were in their early stages at the time of his writing and he never used any of those terms). But, in another memorable phrase, he did make the case for having "differential standards before we convict" [1](p. 300), based on costs and benefits. Moving another step beyond statistical significance testing, we need to consider more than the degree of certainty that there is *some *health hazard, and act based on the expected gains and losses, with or without statistical certainty. 

Hill points this out (in an example sufficiently ill-chosen that it may have contributed to his important message being ignored): "On relatively slight evidence we might decide to restrict the use of a drug for early-morning sickness in pregnant women. If we are wrong in deducing causation from association no great harm will be done. The good lady and the pharmaceutical industry will doubtless survive [1](p. 300)."

Setting aside the impolitic dismissal of women's preferences and the unsupported assertion that there is no great harm at stake (as well as the irony of the popularity, withdrawl, and rehabilitation of the morning sickness drug, Bendectin) the underlying point might be his most important lesson: Policy actions that appear to create a net benefit (on average, considering all costs and benefits) should be taken, even without statistical "proof" of an association, while actions that entail great costs should only be taken with sufficient certainty of substantial benefit.

Hill goes on to strengthen his argument: "On fair evidence we might take action on what appears to be an occupational hazard, e.g. we might change from a probably carcinogenic oil to a noncarcinogenic oil in a limited environment and without too much injustice if we are wrong. But we should need very strong evidence before we made people burn a fuel in their homes that they do not like or stop smoking the cigarettes and eating the fats and sugar that they do like [1](p.300)."

Hill clearly stated that the science and data analysis should not be influenced by what is at stake. But health researchers should recognize that the stakes matter, and incorporate a consideration of them into their work. The alternative to carrying out the policy analysis is to leave the weighing of costs and benefits to an unreliable post-science political process.

The observation that the costs and benefits matter, despite being rather obvious, is frequently – indeed, typically – overlooked in public health discussions. The popular decongestant phenylpropanolamine was banned on weak evidence without regard to the high cost to consumers [37]; dietary recommendations are made without considering absolute benefits, let alone the cost to people of avoiding their favorite foods; and health and safety regulations are tremendously uneven in their cost effectiveness, to cite just three examples. The "policy recommendations" paragraph found in many health research papers sometimes quantifies medical costs, but typically ignores lifestyle, psychological, or productivity costs. It is even rare to find quantification of the absolute aggregate benefit that would result from a policy or behavioral change.

Making a good decision does not depend on having studies with confidence intervals that exclude the null. A best decision can be based on whatever information we have now, and indeed a decision will be made – after all, the decision to maintain the status quo is still a decision [20,38]. Hill offered his clearest condemnation of over-emphasizing statistical significance testing, not when he discussed p-values, but when he concluded by saying: "All scientific work is incomplete – whether it be observational or experimental. All scientific work is liable to be upset or modified by advancing knowledge. That does not confer upon us a freedom to ignore the knowledge we already have, or to postpone the action that it appears to demand at a given time [1](p. 300)."

The pursuit of the low p-value (or confidence interval that excludes the null) leaves our society postponing apparently useful policy choices while we do more research to try to show what we already believe to be true. It also creates the incentive to use dubious methods (e.g., unstated multiple hypothesis testing, choosing models or transforming data to maximize the effect size [39]) in order to squeeze out significant results. Those same methods can be used by those who would prefer to make real causal relationships disappear below the p = .05 horizon. Making the best of the knowledge we have would reduce such temptations. If epidemiologists help empower policy makers to ban an easily-replaced chemical when we believe there is, say, a 50-50 chance that it is a health hazard (based on an honest assessment of all uncertainty), then the payoff for fiddling with the data to show the certainty is a bit higher or a bit lower would be eliminated.

This would release us from the trap of letting ignorance trump knowledge. Regulators often fail to act because we have not yet statistically "proven" an association between an exposure and a disease, even when there is enough evidence to strongly suspect a causal relationship. There is a growing movement to escape this mistake by making a similar mistake in the other direction: adopting precautionary principles, which typically call for restrictions until we have "proven" lack of causal association – a decision based on ignorance that merely reverses the default. If we can escape from the false dichotomy of "proven vs. not proven," facilitated by the nonexistant bright line implied by statistical hypothesis testing and by the notion that causality can be definitively inferred from a list of criteria, then we can make decisions based on what we do know rather than what we don't.

## Conclusions

The uncritical repetition of Hill's "causal criteria" is probably counterproductive in promoting sophisticated understanding of causal inference. But a different list of considerations that can be found in his address is worthy of repeating:

• Statistical significance should not be mistaken for evidence of a substantial association.

• Association does not prove causation (other evidence must be considered).

• Precision should not be mistaken for validity (non-random errors exist).

• Evidence (or belief) that there is a causal relationship is not sufficient to suggest action should be taken.

• Uncertainty about whether there is a causal relationship (or even an association) is not sufficient to suggest action should not be taken.

These points may seem obvious when stated so bluntly, but causal inference and health policy decision making would benefit tremendously if they were considered more carefully and more often. The last point may be the most important unlearned lesson in health decision making.

In fairness to those who do not appreciate these points even today, it overinterprets Hill's short paper to claim that he clearly laid out these considerations, or that he was calling for modern decision analysis and uncertainty quantification. But the fundamental concepts were clearly there (and the overinterpretation is not as great as that required to derive a checklist of criteria for determining causation). Several generations of advancement in epidemiology and policy analysis provide much deeper exposition of his points. But Hill still offers timeless insightful analysis about how to interpret our observations. Strangely, these forgotten lessons, which are only slowly and grudgingly being appreciated in modern epidemiology, are hidden in plain sight, in what is possibly the best known paper in the field.

## Endnotes

1. Interestingly, there are more extreme cases of a scholar's name being immortalized for something contrary to his beliefs. The "Coase Theorem" in economics, from one of the most cited article in the economics and legal literatures [6] (often identified as the most cited article in one of those fields or in their intersection), is usually invoked to make worldly claims that certain beneficial transactions will occur (which, among other things, reduce the need for regulation). But much of Coase's work (including that paper) focuses on how the circumstances required for those transactions to take place are absent in the real world.

2. Reporting confidence intervals provides more information about the estimated association of an exposure and outcome. For example, a large measured effect with a wide confidence interval and a small measured effect with a narrow confidence interval may have the same p-value, but the confidence intervals suggests that a large association is likely in the former case, but not the latter. This has implications for both scientific conclusions and decision making. However, the reporting of confidence intervals addresses only this limitation, not others described subsequently.

3. In effect, Hill claimed that the association was so strong that neither the random nor the systematic error could explain it. In doing so, he failed to heed his own observation that systematic errors might explain an association no matter how low the p-value, and invoked the strength of the statistical association to rule out the possibility it was caused by systematic error. More important, Hill made the mistake of overestimating his ability to intuitively assess complicated quantitative relationships. In Hill's defense, his remark predated the research, primarily from the 1970s and 1980s, that demonstrated that both lay people and experts have poor quantitative intuition (most of the key papers from that literature can be found in a few collected volumes [27-30]). Current researchers who argue that their intuition obviates the need for modern methods for quantifying uncertainty have no such excuse.
